# Identifying long-term effects of SARS-CoV-2 and their association with social determinants of health in a cohort of over one million COVID-19 survivors

**DOI:** 10.1186/s12889-022-14806-1

**Published:** 2022-12-20

**Authors:** Sumit Mukherjee, Meghana Kshirsagar, Nicholas Becker, Yixi Xu, William B. Weeks, Shwetak Patel, Juan Lavista Ferres, Michael L. Jackson

**Affiliations:** 1 Insitro Labs, work done while at Microsoft, South San Francisco, USA; 2 AI for Good Research Lab, Microsoft Corporation, 1 Microsoft Way, WA 98052 Redmond, USA; 3 University of Washington, Seattle, USA; 4 Kaiser Permanente Washington, Seattle, USA

**Keywords:** SARS-CoV-2COVID-19 Long-term effects, Social determinants of health, Medical claims, Infectious diseases, Observational study

## Abstract

**Background:**

Despite an abundance of information on the risk factors of SARS-CoV-2, there have been few US-wide studies of long-term effects. In this paper we analyzed a large medical claims database of US based individuals to identify common long-term effects as well as their associations with various social and medical risk factors.

**Methods:**

The medical claims database was obtained from a prominent US based claims data processing company, namely Change Healthcare. In addition to the claims data, the dataset also consisted of various social determinants of health such as race, income, education level and veteran status of the individuals. A self-controlled cohort design (SCCD) observational study was performed to identify ICD-10 codes whose proportion was significantly increased in the outcome period compared to the control period to identify significant long-term effects. A logistic regression-based association analysis was then performed between identified long-term effects and social determinants of health.

**Results:**

Among the over 1.37 million COVID patients in our datasets we found 36 out of 1724 3-digit ICD-10 codes to be statistically significantly increased in the post-COVID period (*p*-value < 0.05). We also found one combination of ICD-10 codes, corresponding to ‘other anemias’ and ‘hypertension’, that was statistically significantly increased in the post-COVID period (*p*-value < 0.05). Our logistic regression-based association analysis with social determinants of health variables, after adjusting for comorbidities and prior conditions, showed that age and gender were significantly associated with the multiple long-term effects. Race was only associated with ‘other sepsis’, income was only associated with ‘Alopecia areata’ (autoimmune disease causing hair loss), while education level was only associated with ‘Maternal infectious and parasitic diseases’ (*p*-value < 0.05).

**Conclusion:**

We identified several long-term effects of SARS-CoV-2 through a self-controlled study on a cohort of over one million patients. Furthermore, we found that while age and gender are commonly associated with the long-term effects, other social determinants of health such as race, income and education levels have rare or no significant associations.

**Supplementary Information:**

The online version contains supplementary material available at 10.1186/s12889-022-14806-1.

## Background

Since emerging in late 2019, the SARS-CoV-2 virus is known to have infected over 200 million persons globally and caused over 4.5 million deaths as of March, 2022 [1]. SARS-CoV-2 infection can lead to severe primary illness, including pneumonia and acute respiratory distress syndrome [2]. Infection can also lead to numerous immune-mediated pathologies such as lymphopenia during the acute illness phase [3]. Beyond the initial infection, evidence is accumulating that SARS-CoV-2 infection may cause long-term health complications for some individuals [4, 5].

While early studies suggest that SARS-CoV-2 infection can cause multiple long-term complications, much remains unknown about the clinical course following SARS-CoV-2. There are few systematic studies of conditions that may be triggered by infection, and risk factors for long-term SARS-CoV-2 complications on small sample sized cohorts [6–8] or focused on specific conditions [7] While there exist large-scale studies [9] or meta-studies [10], these lack analyses exploring the effects of various social and economic factors, that are known to be powerful determinants of population health.

In this study we utilized claims data from a large sample sized cohort of patients diagnosed with SARS-CoV-2 to study the long-term sequelae arising due to SARS-CoV-2, where long-term sequelae or “long COVID” are defined as symptoms that are observed beyond 2 months after the initial COVID diagnosis. Similar criteria have been used in prior work to define “long COVID” [11, 12]. Our primary contributions are: i) identification of conditions that are significantly more likely to occur after exposure to SARS-CoV-2, ii) identification of the relative timing of when such conditions become significant, iii) identification of the association of significant long-term effects with various social determinants of health (SDOH) such as race, education level, income, etc.

## Methods

### Data source

Our study uses de-identified United States medical claims records from Change Healthcare collected over a period from April 1, 2018, to Jan 31, 2021, encompassing over 50 million records from over 2 million patients. Every claims record contains information about the medical encounter, including the diagnoses, the procedures performed and prescribed drugs. The diagnoses are encoded using International Classification of Diseases, 10th revision (ICD-10) that has diagnosis codes for diseases, signs and symptoms, abnormal findings, injury.

Our claims dataset includes primarily open claims, and a subset of closed payer claims which are normalized for analytics purposes providing sound directional insight for this study. The open claims are derived from broadbased healthcare sources, claims clearing-houses and consist of all medical claims which Change Healthcare processes and for which they have the rights to use. Open claims provide a real-time albeit partial snapshot of the longitudinal journey of a patient. The closed claims are derived from the payer and come from health plans linked at the patient-level and thus capture nearly all events that occur during the patient’s enrollment period. Roughly 95% of the claims used for this study are commercial and 5% are Medicare Advantage/other types of plans.

In addition to medical claims, we use patient-level social determinants of health (SDOH) data from Change Healthcare. The SDOH attributes included in this study are: i) race, ii) gender, iii) age, iii) income, iv) education level, v) veteran status. Of these attributes, gender and age are obtained from patient claims. SDOH data (other than gender and age) are available for 43.91% of the individuals in the data. Race, income, education level and veteran status are missing on roughly 56% of the individuals.

### Study population

Our dataset includes all COVID-19 positive patients, identified by the ICD-10 diagnosis codes of U07.1 (COVID-19, virus identified, lab confirmed) or U07.2 (COVID-19, virus not identified but clinically diagnosed) as the principal diagnosis. These codes are provided by the physicians providing treatment for billing purposes; hence we do not have the information on which clinical features were used at arriving on the diagnosis. We defined a subject’s *index date* as the date of the SARS-CoV-2 diagnosis and only included patients whose index date was between March 1, 2020, and September 30, 2020. For these patients, we had claims data available between April 1, 2018, to January 31, 2021. The total size of our study population was 2.7 million, reduced to 1.37 million after discarding records with missing fields. Of this group, we possess supplementary SDOH data for 602,025 patients. Henceforth, we shall refer to the cohort of patients for whom we possess the SDOH data as the ‘SDOH cohort’ and the other patients as the ‘non-SDOH cohort’. The non-SDOH cohort is used to first define the long-term effects of interest, as described in the statistical analysis section. We then test the association of certain long-term effect outcomes with the SDOH variables using the SDOH cohort. The descriptive statistics of both cohorts can be found in Table 1 and Supplementary Fig. 3 shows a consort diagram for the entire study cohort. We can see that the SDOH and non-SDOH populations are qualitatively similar in terms of age and gender.

**Table 1:** Descriptive statistics of the study cohort

Variable	Category	SDOH fraction	non-SDOH fraction	All fraction
**Age**	0–20	0.009	0.159	0.093
	21–30	0.081	0.118	0.102
	31–40	0.120	0.117	0.118
	41–50	0.149	0.123	0.135
	51–60	0.208	0.153	0.177
	61–70	0.200	0.148	0.171
	71–80	0.145	0.109	0.125
	80+	0.088	0.073	0.079
**Gender**	Female	0.611	0.581	0.594
	Male	0.389	0.419	0.406
**Veteran status**	Non-veteran	0.799	x	x
	Veteran	0.201	x	x
**Race**	Asian	0.028	x	x
	Black	0.120	x	x
	Hispanic	0.190	x	x
	White	0.663	x	x
**Income**	Less than $15,000	0.102	x	x
	$15,000 - $19,999	0.072	x	x
	$20,000 - $29,999	0.106	x	x
	$30,000 - $39,999	0.107	x	x
	$40,000 - $49,999	0.103	x	x
	$50,000 - $74,999	0.202	x	x
	$75,000 - $99,999	0.116	x	x
	$100,000 - $124,999	0.062	x	x
	Greater than $124,999	0.131	x	x
**Education**	Completed High School	0.608	x	x
	Completed College	0.270	x	x
	Completed Graduate School	0.115	x	x
	Attended Vocational/Technical	0.007	x	x

### Study design

We utilize a self-controlled cohort design (SCCD) [13] in this study. In this design, event rates during a time window after SARS-CoV-2 diagnosis are compared to event rates during a time window prior to diagnosis, where the study population is restricted to patients diagnosed with SARS-CoV-2. The outcome period is defined as beginning 2 months after the index date and continuing through January 31, 2021, the last date for which reliable claims data are present (see Supplementary Fig.1). The control period is defined as the three-month period from 10 months to 7 months prior to the index date. This control period begins during the same calendar month as the outcome period, and so should reduce possible confounding by seasonal variations in incidence of events of interest.

We consider all patients with any medical records in the outcome period. This includes out-patient visits, telehealth appointments and in-patient visits (i.e. patients who were hospitalized). Since our data is medical encounter based, milder occurrences of symptoms such as fatigue, headache that do not result in a medical encounter are less likely to appear in the records.

Pre-existing comorbidities were defined based on ICD-10 codes assigned to medical encounters during the six-month period from 16 months to 10 months prior to the index date (see Supplementary Fig. 1). This period does not overlap with the control period, so events during the control period will not also be counted as comorbidities. The Elixhauser comorbidity index [14] was used to define comorbid conditions and their corresponding ICD-10 codes [15].

### Statistical analysis

*Identification of statistically significant ICD10 codes that define long term effects –* Following common practice, we grouped the ICD10 codes by their first three digits which approximately represents high level health conditions. Relative abundances for each condition (represented by a three-digit ICD10 code) were calculated for both control and post-COVID periods. Conditions that occurred in less than 0.01% of the post-COVID population (i.e. in 137 out of the 1.37 million people) were discarded to limit the analysis to conditions that were present in a large enough population. A 2-proportion one-way z-test was performed to identify conditions that were significantly higher in the post-COVID period, compared to the control period. The significance level was set to 0.05 with multiple testing correction using the Bonferroni method, for this and all subsequent analyses unless mentioned otherwise. The resulting *p*-value after correction was 0.0014. This analysis was done on the non-SDOH cohort. We then took the statistically significant codes and checked how significant these are in the SDOH cohort. The results are shown in Table 2 and discussed further in the Results section.

**Table 2:** ICD10 codes that were observed in a significantly higher proportion in the post-COVID window compared to the control window. ICD10 codes that are significant for both non-SDOH and SDOH cohorts are in bold

ICD10	Description	Non-SDOH cohort			SDOH cohort		
		Control%	Post%	p-value	Control%	Post%	p-value
**A41**	Other sepsis	0.667	0.813	2.0E-26	0.684	0.761	3.9E-07
B49	Unspecified mycosis	0.012	0.019	5.0E-05	0.012	0.021	1.3E-04
**B94**	Sequelae of infectious and parasitic diseases	0.002	0.041	3.6E-63	0.001	0.040	1.3E-49
D84	Other immunodeficiencies	0.036	0.052	1.1E-06	0.043	0.058	8.4E-05
**E43**	Severe protein-calorie malnutrition	0.126	0.214	4.8E-40	0.131	0.204	3.5E-23
**E44**	Medium/Mild protein-calorie malnutrition	0.158	0.200	1.8E-10	0.167	0.201	6.7E-06
**E46**	Unspecified protein-calorie malnutrition	0.148	0.232	2.4E-33	0.144	0.224	3.9E-25
**G72**	Unspecified myopathies	0.032	0.107	5.4E-71	0.042	0.144	5.0E-75
G92	Toxic encephalopathy	0.090	0.112	5.0E-06	0.093	0.111	6.9E-04
**G93**	Other disorders of brain	0.702	0.847	5.6E-25	0.702	0.846	8.7E-20
**I26**	Pulmonary embolism	0.206	0.309	1.7E-36	0.293	0.392	4.5E-21
I40	Acute myocarditis	0.002	0.010	2.9E-10	0.002	0.006	5.5E-04
**I46**	Cardiac arrest	0.030	0.099	3.6E-63	0.030	0.095	2.1E-46
I82	Other venous embolism/thrombosis	0.419	0.484	7.2E-10	0.539	0.574	4.9E-03
**J12**	Viral pneumonia	0.055	0.938	0.0E+ 00	0.055	1.110	0.0E+ 00
**J69**	Pneumonitis due to solids and liquids	0.151	0.190	2.3E-09	0.128	0.160	1.4E-06
**J80**	Acute respiratory distress syndrome	0.018	0.127	2.6E-137	0.019	0.137	1.3E-118
J84	Other interstitial pulmonary diseases	0.218	0.276	1.5E-13	0.326	0.362	4.1E-04
J91	Pleural effusion	0.036	0.049	4.6E-05	0.041	0.053	1.3E-03
**J93**	Pneumothorax and air leak	0.036	0.067	7.9E-18	0.040	0.070	5.4E-13
**J95**	Intraoperative/postprocedural complications	0.043	0.072	2.8E-14	0.040	0.072	5.0E-14
**J96**	Respiratory failure	1.065	1.822	0.0E+ 00	1.178	1.910	4.5E-233
**K94**	Complications of artificial openings of the digestive system	0.085	0.114	9.2E-09	0.054	0.093	4.0E-15
L63	Alopecia areata	0.029	0.041	4.0E-05	0.038	0.050	7.7E-04
**L64**	Androgenic alopecia	0.013	0.026	9.1E-09	0.018	0.033	3.4E-07
**L65**	Telogen effluvium	0.116	0.354	3.9E-204	0.142	0.439	5.5E-202
**L89**	Pressure ulcer	0.335	0.605	3.9E-132	0.368	0.674	2.3E-120
M30	Polyarteritis nodosa and related conditions	0.005	0.013	1.1E-07	0.004	0.003	9.0E-01
O98	Maternal infectious and parasitic diseases	0.057	0.079	2.7E-07	0.041	0.045	1.4E-01
**R13**	Aphagia and dysphagia	1.077	1.249	1.8E-23	1.069	1.168	1.1E-07
**R43**	Disturbances of smell and taste	0.026	0.147	1.2E-143	0.040	0.153	2.1E-89
**R57**	Shock	0.052	0.095	1.1E-23	0.054	0.089	1.2E-13
R64	Cachexia	0.032	0.047	3.3E-06	0.033	0.045	4.7E-04
**R65**	Systemic inflammation and infection	0.238	0.320	3.2E-22	0.256	0.304	3.2E-07
**R77**	Other abnormalities of plasma proteins	0.042	0.077	2.2E-19	0.048	0.084	1.3E-14
**R78**	Findings of drugs and other substances, not normally found in blood	0.166	0.207	2.0E-09	0.173	0.212	3.8E-07

*Identification of co-occurring long-term effects –* Identification of frequently co-occurring conditions was done using a data mining technique known as market-basket analysis or affinity analysis [14]. Briefly, affinity analysis identifies co-occurring items (long-term effects in our case) in the data by comparing the observed co-occurrence frequency with the expected co-occurrence frequency (assuming that the co-occurrence was purely random). We first performed market affinity analysis with (support ≥ 0.01, lift ≥ 1) on the post-COVID period to identify co-occurring conditions. We then identified the relative proportion of patients who experienced each ‘basket’ of conditions in the post-COVID and control periods. Finally, we performed a 2-proportion one-way z-test to identify which baskets were significantly overrepresented in the post-COVID period compared to the control period. This analysis was performed on the non-SDOH cohort.

*Identification of month-wise long-term effects –* To study the month-wise prevalence of the long-term effects that we identified, we perform the same analysis as described in the previous section, on one month long post-COVID and matched control periods shown in Supplementary Fig. 1. The analysis was done for months 3, 4 and 5 post-COVID. Since we had used the non-SDOH cohort to identify the long-term effects, to prevent ‘double dipping’, we performed this analysis on the SDOH cohort.

*Studying associations of SDOH variables with long-term effects* – Association testing of SDOH variables with each significant long-term effect was done using a logistic regression model, which adjusted for comorbidities and presence of the same long-term conditions in the control period (prior events). The mathematical model can be expressed as:

$$\log \left(\frac{p_m}{1-{p}_m}\right)={\beta}_0+\sum_{i\in SDOH}{\beta}_i^{SDOH}{X}_i+\sum_{j\in Comorb}{\beta}_j^{Comorb}{X}_j+\sum_{k\in PriorEvents}{\beta}_k^{PriorEvents}{X}_k$$

Where, *p*_*m*_ = Pr(*Y*_*m*_ = 1) is the probability of long-term effect *m* occurring. Prior to performing the logistic regression, we performed feature selection using a chi-squared test of independence between each outcome and independent variable. We use statsmodel (LBFGS for optimization) for learning the logistic regression parameters: beta. Cross-validation was not performed as the main purpose of the logistic regression model is to understand the association between the SDOH variables and long term effects of interest. In addition, the sample size is much larger compared to the dimension of input. As a result, overfitting is not a concern in this case.

Only variables that met a significance level of 0.05 were used in the logistic regression. However, a Bonferroni corrected *p*-value (correcting for *m* outcomes, the resultant p-value threshold after correction was 0.0014) was used to determine significant associations in the logistic regression model. The selected baseline categories were: Race-White, Education – Completed college, Income-greater than $124,999, Gender-male, Non-veteran, Age-31-40.

## Results

### Long term effects of COVID-19 and cooccurring conditions

The study population consisted of 1,371,110 patients with an ICD-10 diagnosis code for SARS-CoV-2 infection. This population was predominantly older (mean age 55.36 years, standard deviation 17.6 years) and female (59.44%). Among the 43.91% of the cohort with SDOH data available, 66% were non-Hispanic White.

Of the 1724 3-digit ICD10 codes considered, 36 met the significance threshold after the Bonferroni correction (resultant *p*-value threshold after correction was 0.0014). These ICD10 codes are reported in Table 2, along with the rate of occurrence in the control and post-COVID period for the non-SDOH and SDOH cohorts. The identification of significant ICD10 codes was done using the *p*-values from the non-SDOH cohort only and the SDOH cohort numbers are only reported for validation purposes. We find that all 36 were at least nominally significant and 32 codes have a *p*-value < 0.05 and, after multiple testing correction, 25 of 36 ICD10 codes are significant in both cohorts, thereby indicating the consistency of our findings. We do not use the SDOH cohort for both the selection of the codes and for SDOH-association analysis, since the results will be biased due to the lack of a held-out population and result in finding spurious associations.

Several broad categories of associations are notable. Unsurprisingly, multiple codes suggest ongoing pulmonary complications, such as J12 (viral pneumonia) and J80 (acute respiratory distress syndrome). Cardiac and thrombotic events comprise a second category (e.g. I40 [acute myocarditis], I82 (other venous embolism and thrombosis). A third category is apparent complications of treatment during acute SARS-CoV-2 infection, such as codes J95 (intraoperative and postprocedural complications), K94 (complications of artificial openings of the digestive system), and L89 (pressure ulcer). A fourth is malnutrition or wasting, such as codes E43, E44, and E46 (protein-calorie malnutrition) or R34 (cachexia).

Next, we investigated two of the significant 3-digit digit codes: D84 (other immunodeficiencies) and G93 (other disorders of brain). We evaluated whether the significant associations with these codes were driven by specific sub-codes. For these two ICD-10 code families, we identified the constituent ICD-10 codes that were significantly increased in the post-COVID period compared to the control period using the same method as above. However, unlike the previous analysis, here we only look at the SDOH cohort, since the significant 3-digit codes were identified on the non-SDOH cohort. To avoid overstating the significance, we focus on the SDOH cohort, since the significant 3-digit codes were identified using the non-SDOH cohort.

Of the 19 constituent codes, we find 5 that meet our Bonferroni corrected significance threshold (see Supplementary Table 1). The only significant sub-code to D89 was D89.9 (immunodeficiency, unspecified). Four sub-codes were significant from G93. Of these, the most significant was G93.3 (postviral fatigue syndrome), which was 4.4 times more common in the post-COVID period than the pre-COVID period.

Using association analysis on the post-COVID period, we identified several co-occurring long-term conditions. We then looked at the co-occurring conditions that are significantly overrepresented in the post-COVID period compared to the control period (see Supplementary Table 2) after excluding those that contained Z codes. We exclude Z codes as they do not reflect medical conditions and generally represent healthcare events. Z codes are also rarely used [16], (< 1% of the population having Z codes available) which can introduce a bias in the analysis.

Only one co-occurring condition was found to meet our significance threshold: D64 (other anemias) and I10 (essential hypertension). This condition was also found to be significant in the SDOH cohort.

### Persistent and fleeting long term effects

We explored the timing of significant associations at the 3-digit code level, within one-month windows during the long-COVID phase (Table 3). Several code groups were significantly elevated early on but appeared to have resolved by month 5 post-onset, while others were elevated through the full follow-up period. Of the categories of codes identified earlier, no category saw resolution of all codes by 5 months post-onset, and no category saw persistence of all codes through 5 months. Only L64 (androgenic alopecia) became significantly elevated at month 5 after not being elevated previously.

**Table 3:** ICD10 codes that are significantly over-present in the 1 month long post-COVID periods compared to corresponding month-long control period

ICD10		Month 3	Month 4	Month 5
D84	Other immunodeficiencies	No	No	No
L63	Alopecia areata	No	No	No
B49	Unspecified mycosis	Yes	No	No
G92	Toxic encephalopathy	Yes	No	No
I82	Other venous embolism/thrombosis	Yes	No	No
J69	Pneumonitis due to solids and liquids	Yes	No	No
J91	Pleural effusion	Yes	No	No
K94	Complications of artificial openings of the digestive system	Yes	No	No
M30	Polyarteritis nodosa and related conditions	Yes	No	No
O98	Maternal infectious and parasitic diseases	Yes	No	No
R78	Findings of drugs and other substances, not normally found in blood	Yes	No	No
L64	Androgenic alopecia	No	No	Yes
A41	Other sepsis	Yes	Yes	No
E44	Medium/Mild protein-calorie malnutrition	Yes	Yes	No
G93	Other disorders of brain	Yes	Yes	No
I40	Acute myocarditis	Yes	Yes	No
J84	Other interstitial pulmonary diseases	Yes	Yes	No
J93	Pneumothorax and air leak	Yes	Yes	No
J95	Intraoperative/postprocedural complications	Yes	Yes	No
R13	Aphagia and dysphagia	Yes	Yes	No
R57	Shock	Yes	Yes	No
R64	Cachexia	Yes	Yes	No
R65	Systemic inflammation and infection	Yes	Yes	No
R77	Other abnormalities of plasma proteins	Yes	No	Yes
B94	Sequelae of infectious and parasitic diseases	Yes	Yes	Yes
E43	Severe protein-calorie malnutrition	Yes	Yes	Yes
E46	Unspecified protein-calorie malnutrition	Yes	Yes	Yes
G72	Unspecified myopathies	Yes	Yes	Yes
I26	Pulmonary embolism	Yes	Yes	Yes
I46	Cardiac arrest	Yes	Yes	Yes
J12	Viral pneumonia	Yes	Yes	Yes
J80	Acute respiratory distress syndrome	Yes	Yes	Yes
J96	Respiratory failure	Yes	Yes	Yes
L65	Telogen effluvium	Yes	Yes	Yes
L89	Pressure ulcer	Yes	Yes	Yes
R43	Disturbances of smell and taste	Yes	Yes	Yes

### Associations with SDOH variables

**Fig. 1:**
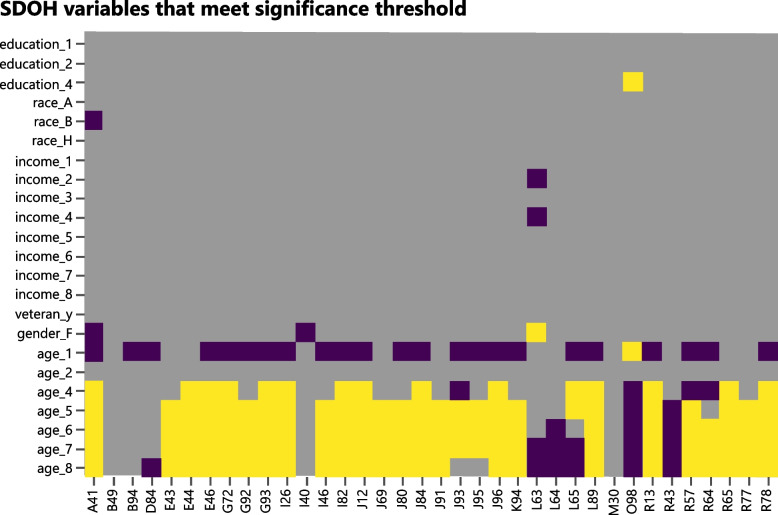
Association analysis of SDOH variables with ICD10 codes corresponding to long term effects of COVID-19. Green indicates no association, blue indicates a negative association, yellow indicates a positive association

As described in the Methods section, we estimated the association of every significant 3-digit code group with the SDOH variables, adjusting for comorbidities and the presence of the same conditions in the control period (Fig. 1). Interestingly, for this population, race was only significantly associated with A41 (other sepsis). Similarly, only L63 (alopecia areata) was significantly associated with some income categories. Since the evidence on ‘alopecia areata’ being a long-term consequence after COVID-19 infection is limited [17], we do not explore this association with income further.

Female gender showed negative association with A41, I40 and positive association with L63. In contrast, age was significantly associated with most of the long-term effect conditions. A complete table of association results for SDOH, as well as comorbidity and prior conditions can be found in Supplementary Table 2.

## Discussion

Long-term sequelae of SARS-CoV-2 infection has received substantial attention in scientific literature, legacy media, and social media. Numerous studies have explored the long-term health concerns among people previously infected with SARS-CoV-2 [4, 5, 8–10, 18, 19]. However, some prior research has been limited by small sample sizes [18, 19] or lack of comparison groups [19] or based on self-reported symptoms [11] and large-scale studies such as OpenSAFELY [9] have lacked an analysis of socio-demographic factors. This study addresses these limitations by comparing the frequency of ICD-10-coded diagnoses during pre- and post-COVID-19 periods among more than 1.37 million study subjects.

Our first notable finding was that SARS-CoV-2 infection was associated with subsequent codes for malnutrition/wasting. This accords with a prior cohort study that found 31% of patients hospitalized for COVID-19 lost ≥5% of their body weight at roughly 3 weeks post-discharge compared to admission body weight [18]. Malnutrition is known to be a risk factor for pneumonia in diverse populations, including community-dwelling seniors [20], seniors in long-term care settings [21] and children in resource-poor settings [22]. COVID-19-induced malnutrition/wasting thus may pre-dispose patients to future episodes of pneumonia or other respiratory disease.

A second notable finding is the frequency of codes likely related to complications of COVID-19 or hospitalization due to severe disease. These include conditions such as pressure ulcers and enterostomy. Please note that not all patients in our dataset were hospitalized – ~ 88% were not hospitalized and 12% had hospital stays, with a median hospital stay of 4 days amongst the hospitalized population. Even among those successfully treated for COVID-19, hospitalization and treatment can have long-term impacts on health, independent of physiologic damage caused by infection.

Third, the ICD-10 codes associated with SARS-CoV-2 infection in this study support many self-reported complications from surveys of COVID-19 patients. Post-viral fatigue syndrome (G93.3) was significantly associated with SARS-CoV-2 infection in this study. This matches patient-reported data, where fatigue is commonly reported [19]. Other codes that match common patient-reported outcomes include persistent respiratory symptoms, myalgia, and ongoing disturbances to taste/smell.

We also found that symptoms seemed to vary, over time, with a number of symptoms no longer being over-represented as compared to a year earlier as time progressed. This suggests that some post-COVID symptoms may mitigate over time. Longer term studies are required to determine the ultimate persistence of particular post-COVID symptoms.

Interestingly, some post-COVID-19 complications frequently reported by patients did not show up in the ICD-10 data. Examples include headache, anxiety, and sleep disturbances. It could be that these complications are frequently experienced by patients but do not result in medical encounters; or that other, more severe symptoms ended up in the ICD-10 coded data instead; or that these symptoms are actually not elevated among persons infected with SARS-CoV-2 relative to pre-infection periods. Further research will be needed to distinguish between these possibilities.

One recent study by Murk et al. [26] applied a similar design to medical claims data to identify short-term (< 31 days) complications of SARS-CoV-2 infection [4]. Like our study, that study found elevated risks of codes associated with respiratory infection and respiratory complications, disturbance of taste/smell, and cardiovascular conditions such as cardiac arrest. The most notable difference is that Murk and colleagues found associations with acute kidney failure, which was not observed in the present study.

Finally, we found very few socio-demographic variables that were associated with persistent post-COVID symptoms: for most, older age group was associated with higher persistence. This is consistent with a multitude of papers that have found older age to be a major predictor for COVID severity [23]. It is possible that age so dominated the models that we used that other variables did not significantly contribute to the model.

Several limitations of this study are important to highlight. First, the self-controlled cohort design assumes that differences in event frequencies after vs. before SARS-CoV-2 diagnosis are causally related to infection. Other temporal trends in these diagnoses unrelated to infection could bias effect estimates either upward or downward. Second, this study relies on ICD-10 codes assigned to medical encounters. ICD-10 codes are imperfect proxies for actual disease and do not allow evaluation of complications that are not severe enough to warrant medical attention. These codes also do not include indicators of disease severity. Third, since a majority of our dataset consists of open claims, we do not have a complete record of all the encounters for every patient. Hence, the statistics we derive at the population-level are reliable, but we are unable to perform patient-level prediction. Fourth, the patients come from commercial insured population of patients, and thereby represent largely healthy individuals and are likely to miss individuals with the most adverse outcomes, such as those on Medicare plans. Finally, SDOH data were only available for 43.91% of our population. This data may not be missing at random, and the group with SDOH data may not be representative of the underlying population. SDOH associations should thus be interpreted with caution. It is possible that some ICD-10 codes that were only present in the SDOH cohort are missed by our approach that finds significant codes using the non-SDOH cohort.

## Conclusions

In this study, we have identified potential complications of SARS-CoV-2 infection that require ongoing medical evaluation and care. This builds on and supplements patient-reported outcomes and illustrates the potential for long-term complications of SARS-CoV-2 infection. Furthermore, we find that after controlling for prior health conditions, only age and gender consistently show significant associations with the identified long-term effects.

## Supplementary Information

**Additional file 1:** **Supplementary Figure 1.** Study design showing the time periods for defining the co-morbidities, the outcome and control periods with respect to the COVID-19 onset date. A) Study design for identification of long-term effects and their association with SDOH variables. B) Study design for identification of long-term effects in different one-month windows post-diagnosis. **Supplementary Figure 2.** Association analysis of comorbidity and past conditions with ICD10 codes corresponding to long term effects of COVID-19. Green indicates no association, blue indicates a negative association, yellow indicates a positive association. **Supplementary Figure 3.** CONSORT diagram showing the cohort. **Supplementary Table 1.** 4 digit ICD10 codes (in the D84.* and G93.* range) that were observed in a significantly higher proportion in the post-covid window compared to the control window. **Supplementary Table 2.** Co-occurring patterns whose presence was significantly higher in the post-COVID period compared to the control period. Reported statistics and p-values computed on the non-SDOH cohort.

## Data Availability

The data that support the findings of this study are available from Change Healthcare but restrictions apply to the availability of these data, which were used under license for the current study, and so are not publicly available. Data are however available from the authors (please contact Nicholas Becker: Nicholas.Becker@microsoft.com) upon reasonable request and with permission of Change Healthcare.
