# Supplementary material for: Identifying long-term effects of SARS-CoV-2 and their association with social determinants of health in a cohort of over one million COVID-19 survivors
Source: BMC Public Health. 2022 Dec 20;22:2394. doi: 10.1186/s12889-022-14806-1 (PMC9765366; doi:10.1186/s12889-022-14806-1)
Supplement: Supplementary file 1 — Additional file 1: Supplementary Figure 1. Study design showing the time periods for defining the co-morbidities, the outcome and control periods with respect to the COVID-19 onset date. A) Study design for identification of long-term effects and their association with SDOH variables. B) Study design for identification of long-term effects in different one-month windows post-diagnosis. Supplementary Figure 2. Association analysis of comorbidity and past conditions with ICD10 codes corresponding to long term effects of COVID-19. Green indicates no association, blue indicates a negative association, yellow indicates a positive association. Supplementary Figure 3. CONSORT diagram showing the cohort. Supplementary Table 1. 4 digit ICD10 codes (in the D84.* and G93.* range) that were observed in a significantly higher proportion in the post-covid window compared to the control window. Supplementary Table 2. Co-occurring patterns whose presence was significantly higher in the post-COVID period compared to the control period. Reported statistics and p-values computed on the non-SDOH cohort. [file 12889_2022_14806_MOESM1_ESM.docx]

Supplementary Figures/Tables: Identifying long-term effects of SARS-CoV-2 and their association with social determinants of health in a cohort of over one million COVID-19 survivors

Authors: Sumit Mukherjee^5^* (BS, MS, PHD), Meghana Kshirsagar^1^* (BS, MS, PHD), Nicholas Becker^1,2^ (BS, MS), Yixi Xu^1^ (BS, PHD), William B Weeks^3^ (BS, MD, MBA, PHD), Shwetak Patel^2^ (BS, PHD), Juan Lavista Ferres^1^ (BS, MS), Michael L. Jackson^4^ (BA, MPH, PHD).

*Equal contributing authors in random order of names

^1^AI for Good Research Lab, Microsoft Corporation, Redmond, USA.

^2^University of Washington, Seattle, USA.

^3^Microsoft Corporation, Paris, France.

^4^Kaiser Permanente Washington, Seattle, USA.

^5^Insitro Labs, USA

Corresponding author: Dr. Meghana Kshirsagar, email: [Meghana.Kshirsagar@microsoft.com](mailto:Meghana.Kshirsagar@microsoft.com), postal address: 1 Microsoft Way, Redmond, WA 98052, phone: +1 (425) 4218258.

## Contents

Supplementary Figure 1: Study design showing the time periods for defining the co-morbidities, the outcome and control periods with respect to the COVID-19 onset date. A) Study design for identification of long-term effects and their association with SDOH variables. B) Study design for identification of long-term effects in different one-month windows post-diagnosis.

Supplementary Table 1: 4 digit ICD10 codes (in the D84.* and G93.* range) that were observed in a significantly higher proportion in the post-covid window compared to the control window.

Supplementary Table 2: Co-occurring patterns whose presence was significantly higher in the post-COVID period compared to the control period. Reported statistics and p-values computed on the non-SDOH cohort.

Supplementary Figure 2: Association analysis of comorbidity and past conditions with ICD10 codes corresponding to long term effects of COVID-19. Green indicates no association, blue indicates a negative association, yellow indicates a positive association.

Supplementary Figure 3: CONSORT diagram showing the cohort


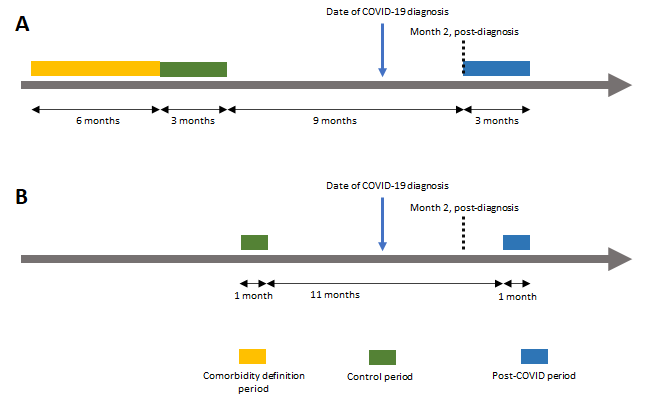


**Supplementary Figure 1: Study design showing the time periods for defining the co-morbidities, the outcome and control periods with respect to the COVID-19 onset date. A) Study design for identification of long-term effects and their association with SDOH variables. B) Study design for identification of long-term effects in different one-month windows post-diagnosis.**

| ICD10 | Condition | Control% | Post% | p-value |
| --- | --- | --- | --- | --- |
| D84.9 | Immunodeficiency | 0.029 | 0.047 | 3.11E-09 |
| G93.1 | Anoxic brain damage | 0.033 | 0.050 | 6.20E-08 |
| G93.3 | Postviral fatigue syndrome | 0.015 | 0.066 | 1.92E-55 |
| G93.4 | Unspecified encephalopathy | 0.492 | 0.605 | 9.03E-22 |
| G93.6 | Cerebral edema | 0.020 | 0.028 | 2.79E-04 |

**Supplementary Table 1: 4 digit ICD10 codes (in the D84.* and G93.* range) that were observed in a significantly higher proportion in the post-covid window compared to the control window.**

| ICD10 | Control% | Post% | p-value |
| --- | --- | --- | --- |
| D64, I10 | 1.126794 | 1.15878 | 3.1E-02 |

**Supplementary Table 2: Co-occurring patterns whose presence was significantly higher in the post-COVID period compared to the control period. Reported statistics and p-values computed on the non-SDOH cohort.**

**Supplementary Figure 2: Association analysis of comorbidity and past conditions with ICD10 codes corresponding to long term effects of COVID-19. Green indicates no association, blue indicates a negative association, yellow indicates a positive association.**

**
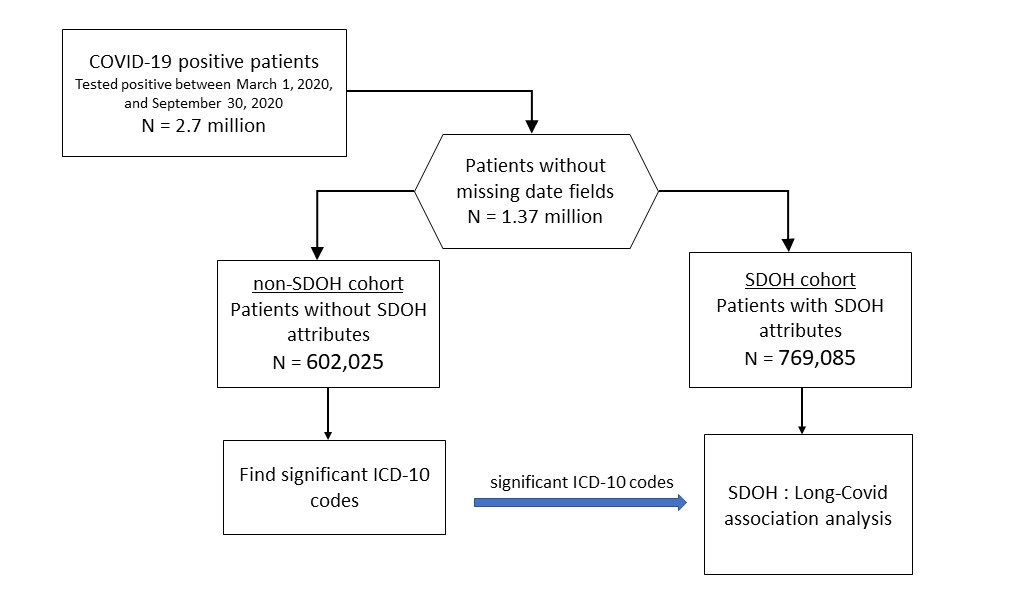
**

**Supplementary Figure 3: FLOW diagram showing the study cohort**
